# Catechins within the Biopolymer Matrix—Design Concepts and Bioactivity Prospects

**DOI:** 10.3390/antiox9121180

**Published:** 2020-11-26

**Authors:** Zvezdelina Yaneva, Donika Ivanova

**Affiliations:** Chemistry Unit, Department of Pharmacology, Animal Physiology and Physiological Chemistry, Faculty of Veterinary Medicine, Trakia University, Students Campus, 6000 Stara Zagora, Bulgaria; donika.ivanova@trakia-uni.bg

**Keywords:** catechins, biopolymer formulations, encapsulation, release, biological activity

## Abstract

Epidemiological studies and clinical investigations proposed that catechins extracts alone may not provide a sufficient level of bioactivities and promising therapeutic effects to achieve health benefits due to a number of constraints related to poor oral absorption, limited bioavailability, sensitivity to oxidation, etc. Modern scientific studies have reported numerous techniques for the design of micro- and nano-bio-delivery systems as novel and promising strategies to overcome these obstacles and to enhance catechins’ therapeutic activity. The objective assessment of their benefits, however, requires a critical comparative estimation of the advantages and disadvantages of the designed catechins-biocarrier systems, their biological activities and safety administration aspects. In this respect, the present review objectively outlines, compares and assesses the recent advances related to newly developed design concepts of catechins’ encapsulation into various biopolymer carriers and their release behaviour, with a special emphasis on the specific physiological biofunctionalities of the innovative bioflavonoid/biopolymer delivery systems.

## 1. Introduction

Modern medicine faces an enormous number of challenges related not solely to the choice of the most appropriate therapy but to the proper selection of the most efficient, human- and eco-friendly therapeutic agents among thousands of conventional drugs, millions of food additives and alternative pharmaceutical formulations. Nowadays, the global pharmaceutical market is flooded with nutraceutical supplements and dietary additives claiming to be of natural origin and sold with a therapeutic claim of “superfood”, but a great deal of them circumvent the European and US laws, regulations, policies and procedures for drug applications [1]. Up to date, the number of green tea formulations and supplements with “incredible” health benefits offered online are over 3000. However, a number of questions related to their application arise: Is their sale regulated? Are there any side effects? Does their real content correspond to the description? Are their health benefits scientifically and clinically proven? Thus, science, pharmacy and alternative medicine are challenged by traditional medicine to prove and accept or refute the biomedical activity and efficiency of nutraceuticals.

**Figure 1:**
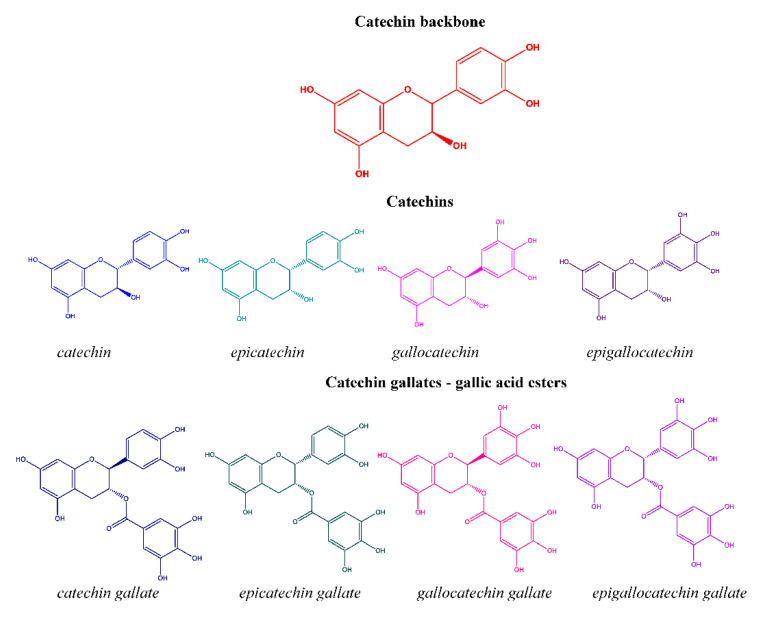
Structural formulas of the catechin family.

Scientific literature presents a great number of studies on the bioactivity and major health benefits of naturally derived catechins (Figure 1). However, today, the major concern of their direct administration as therapeutic agents arises from the proven limitations associated with low bioavailability, poor solubility in the gastrointestinal fluids, sensitivity to oxidation, incomplete absorption from the gastrointestinal tract, reduced or no biological activity and even toxicity [2]. In order to address this issue, principles and techniques of micro- and nano-technology have been constantly utilized with the aim to sustain and enhance the biological activity of catechins, to achieve sustained delivery in a controlled manner [3], to provide protection against harsh acidic physiological environment and to reduce toxicity by combining the bioflavonoids health benefits with the unique features and remarkable physical, chemical and biological properties and functionalities (biocompatibility, biodegradability, antimicrobial, antifungal, analgesic, antitumor activities) of biopolymer materials [4].

Innovative formulation technologies such as: submicron fibres, polymeric and lipid micro- and nano-particles, liposomes, emulsions, polymeric micelles, nanocomposites and nanocomplexes, are currently under thorough research. They are subjected to elimination of catechins limitations and the achievement of improved solubility, increased bioavailability, along with reduced gastric irritation, enhanced permeation through the blood–brain barrier, prevention from oxidation and enzymatic degradation [5]. To address the challenge of the design of efficient bioflavonoids/biopolymer carrier systems and the elucidation of their in vivo behaviour and biological activity, it is necessary to study in detail the chemical structure, properties and the host–guest interactions of the encapsulated substance and the carrier matrix in physiological media, as well as the biomedical functionalities, associated with antibacterial, antiproliferative and antioxidant activities [6,7,8,9,10].

The aim of the present review was to objectively outline, to compare and to assess the recent advances related to newly developed design concepts of catechins encapsulation into various biopolymer carriers and their release behaviour with a special emphasis on the specific physiological biofunctionalities of the innovative bioflavonoid/biopolymer delivery systems.

## 2. Catechins Encapsulation Methodologies

**Figure 2:**
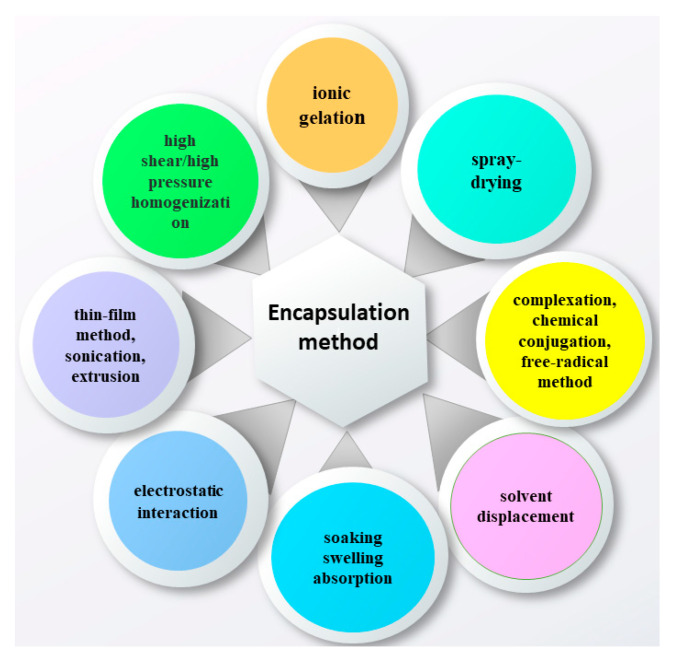
Methodologies for catechins encapsulation into biopolymer formulations.

Recent scientific investigations approved a variety of encapsulation techniques of catechins into various nano- and micro-formulations with biopolymer nature (Figure 2). The mechanism (physical incorporation, electrostatic attraction, chemical reaction, etc.) of the bioflavonoids entrapment in the specific matrix renders significant influence on the physicochemical properties of the carrier systems, and on the in vitro release behaviour. Besides, the encapsulation methodology could affect the bioavailability, stability, stimuli-responsiveness and bioactivity of the released biologically active compound and/or impact on the synergic action of the composite system [11,12,13]. The properties of biopolymer formulations in terms of stability, droplet size, bulk and interfacial viscosity, combined with the evaluation of the properties of the produced particles—morphology, size, encapsulation efficiency of catechins and the stability of the bioflavonoids within the particles under different storage and physiological conditions (humidity, pH and temperature), have to be studied and analysed [13,14]. Thus, the comparative analyses of the pros and cons of encapsulation methodology are essential for the design, synthesis of catechin-carrier systems, the assessment of their bioactivity and objective evaluation of the potential for pharmaceutical and medical applications based on in-depth clinical research.

### 2.1. Ionic Gelation

Ionic gelation, based on the capability of polyelectrolytes to crosslink in the presence of counter ions, is among the most commonly used and well-described techniques of biopolymer formulations’ synthesis [4,5,15,16]. Although earlier studies established some disadvantages of microparticles obtained by this method, such as low encapsulation efficiency of water-soluble drugs, insufficient production yield and presence of organic solvent residues, recent investigations on catechins’ encapsulation displayed the efficiency and increased bioactivity of catechin-loaded formulations newly-synthesized by ionic gelation [17,18,19,20].

Folate-conjugated chitosan nanoparticles for catechin incorporation were synthesized through ionic gelation reactions between folate-modified chitosan and Na-tripolyphosphate. The biopolymer particles were near-spherical in shape. Their bioflavonoid loading capacity was in the range 8.66–11.53%, while the entrapment efficiency reached 19.02–43.28% [7]. Zhang et al. [21] used β-chitosan nanoparticles of different sizes for catechins and catechin-Zn complex loading by ionic gelation technology. The β-chitosan particles with encapsulated catechin-Zn complex were characterized with 208–591 nm particle size, average polydispersity index of 0.386 and positive zeta-potential. The parameter polydispersity index estimates the average uniformity of a particle solution. It is established that higher values correspond to larger size distribution in the particle sample. It is also indicative of particles’ aggregation along with the consistency and efficiency of particle surface modifications. Monodispersed systems usually characterize with polydispersity index < 0.1. Biopolymers’ formulations charge is among the main factors determining their physical stability in physiological medium. High zeta potential values (>20 mV) imply highly charged particles, which prevents aggregation of the particles due to electric repulsion forces prevailing and ensures higher stability of the system. Low values (<0 mV) of the parameter are indicative of attraction forces exceeding repulsion interactions, which could provoke coagulation of the system. However, it should be considered that zeta potential values are not an absolute measurement of nanoparticle stability and the boundary values of the parameter vary in dependence on the nature of the biopolymer formulations and the medium.

An inversely proportional relationship between particle size and antibacterial activity against *Listeria innocua* and *Escharichia coli* was established [21]. The latter observation provided evidence that smaller sized particles exhibited higher antibacterial activity, probably due to the fact that increased surface area ensures greater contact area for mass transfer, as well as a higher extent of bioflavonoid release. Novel chitosan-based nanoparticles with ellipsoidal shape, average diameter of 180.4 nm and zeta potential of 31.79 ± 1.28 mV were synthesized through ionic gelation reaction between chitosan and sodium tripolyphosphate, and subsequently modified by genipin. They displayed enviable entrapment efficiency of approximately 76.35% towards catechin [22]. The study of Lee et al. [23], which compared the physicochemical characteristics and antioxidant activity of catechin-loaded calcium pectinate gel beads, prepared by internal and external gelation, established higher encapsulation efficiency and slower in vitro release of the bioflavonoid from the carrier. The probable explanation of such behaviour could be attributed to the formation of formulations with bioflavonoid-rich core, as a result of the internal gelation procedure, which assures lower rate of release. Whilst, the concentration of encapsulated catechin molecules in the outer coverage of the pectinate beads, obtained by external gelation method, is higher. This results in higher release rate due to the lower resistance, which the organic molecules have to overcome during their desorption from the polymer carrier. However, the synthesis conditions, including catechin/pectin ratio, gelling time, pectin concentration and acetic acid concentration, significantly influenced the entrapment capacity and in vitro release rate of catechin in simulated gastric and intestinal fluids [23].

### 2.2. Electrospinning

Electrospinning is an efficient, facile and versatile technique for producing continuous nanofibers with diameters ranging from a few microns to several nanometres in the form of porous membranes or fibres. It is performed by applying an electrical field to continuously draw the droplet of polymer solution or melt polymer into a fine fibre, followed by its deposition on a grounded collector. The key advantage of the electrospinning process is the absence of heat, which is important for preserving the structure and for achieving high encapsulation efficacy of the bioactive substances upon encapsulation [24,25]. Due to many attractive properties such as large surface-to-volume ratio, porous structure, interconnectivity of pores and the possibility to safely encapsulate active compounds, nanofibers have received great attention in the last years. The encapsulation of therapeutic substances within electrospun fibres allows for adapted and controlled release kinetics, including application at the site of action. These unique properties propose them as candidates for a variety of applications, especially as drug delivery systems and scaffolds for tissue engineering [26,27].

The recent studies of Hoseyni et al. [28,29] performed nanoencapsulation of catechin in a polymeric matrix of Azivash (*Corchorus olitorius.* L) gum-polyvinyl alcohol via electrospinning. It was established that catechin improved the thermal resistance of the nanofibers due to H-bonds interactions with the polymer solution and increased adhesion between molecular chains. The observed in vitro release efficiency was approximately 50% in simulated gastric fluid and about 15% in simulated intestinal medium. Besides, the scientific investigations approved two times slower release rate in high-fat food simulant than in low-fat food simulant. The latter deviation was attributed to the different polarity of both media. The results of this study outline the application of the proposed electrospinning process for the design of active food packaging for improving the oxidative stability of food and pharmaceutical products [28,29]. Zemljic et al. [30] designed electrospun composite nanofibrous material by a combined technique of embedding catechin and resveratrol into chitosan particles via ionic gelation procedure, and further electrospinning for nanofiber formation. Electrospun samples with incorporated (poly)-phenolic component indicated 90% antioxidant activity, compared to samples without active substances, showing inhibition on 2,2′-azino-bis(3-ethylbenzothiazoline-6-sulfonic acid) (ABTS) radicals below 40%. The prepared nano (coated)-functionalised composites (chitosan particles with/without (poly)-phenolic substances) exhibited antimicrobial properties inhibiting the growth of *Escherichia coli* and *Staphylococcus aureus*, by 99% and 83%, respectively. The sustained in vitro release of catechin and the observed synergism between satisfactory antimicrobial properties and high antioxidant efficiency indicated the potential of the nanofiber composites for the development of innovative textiles in the field of healing and treatment of skin wounds [30].

### 2.3. Solvent Displacement Method

The solvent displacement method, known also as the “ouzo” effect, spontaneous emulsification, solvent shifting or non-solvent coacervation, is an emulsification process requiring low energy and no surfactant. Thus, it is applied as an advantageous and efficient alternative technique for nano-/micro-particles fabrication [31]. Pool et al. synthesized pH-sensitive nanoparticles and nanospheres using the biocompatible poly(D,L-lactide-coglycolide) [32] and polymethacrylic acid-co-ethyl acrylate [33] copolymers to study the encapsulation, protection and in vitro release of catechin. The nanoparticles obtained were relatively small (d ≈ 400 nm for poly(D,L-lactide-coglycolide), d ≈ 160 nm for polymethacrylate-based). The entrapped catechin was in an amorphous state with encapsulation efficiency of 79% [34] and 51% [35], respectively. The high negative value of the ζ charge of the nano-formulations proved their stability to aggregation under physiological conditions.

### 2.4. Spray-Drying and Homogenisation

Various modifications of spray-drying and homogenisation methodologies are characterised as rapid, continuous, reproducible and scalable techniques for the design of biopolymer formulations. The synthesis of microspheres with low moisture content, increased production yield and high loading are among their major advantages [34]. A two-step process of homogenisation and spray-drying was successfully applied for the synthesis of spherical, corrugated and polydisperse gum arabic–maltodextrin particles, loaded with epigallocatechin gallate (EGCG). The attenuated total reflection-infrared spectroscopy (ATR-IR) analyses proved the ability of the carbohydrate matrix to preserve the antioxidant activity of EGCG, as well as the significant role of intermolecular interactions in the maintenance of the chemical integrity [35]. The promising potential of the emulsion electro-spraying technology was established by the study of Paximada et al. [36] who encapsulated hydrophilic and lipophilised EGCG into nanoemulsions of spherical sub-micron protein particles with bacterial cellulose as a shell material. The highest encapsulation efficiency (≈97%) of EGCG was achieved by ultrasonic homogenization [36]. A two-step process, involving high-shear and high-pressure homogenisation, was applied for the encapsulation of catechins by lipid-based highly stable nanoemulsion delivery systems, synthesised by sunflower and palm oils and combined with hydrophilic and lipophilic emulsifiers. The carrier systems were characterised with constant droplet diameter, conductivity, refractive index and pH, up to 14 days [37]. An innovative thin-film method with subsequent sonication and extrusion was applied for the production of elastic nanoliposomes by soy phosphatidylcholine, cholesterol and Tween. The formulations were stable against aggregation (zeta potential −15 mV), with high catechin loading (>80%) and diameter of d = 35–70 nm [38].

### 2.5. Chemical Synthesis Methods

Novel modified chemical synthesis methods based on intermolecular and intramolecular interactions, complexation, polymerisation, electrophilic and free radical reactions, conjugation, etc., offer a wide spectrum of possibilities for the design and production of a great variety of catechins-loaded micro- and nano-formulations with unique structural and physicochemical functionalities and therapeutic activity [8,11,39,40,41]. Cheng et al. [39] constructed a catechin-based polyion complex micelle through electrostatic interaction and phenylboronic acid–catechol interaction between poly(ethylene glycol)-block-poly(lysine-co-lysine-phenylboronic acid) and (−)-epigallocatechin-3-O-gallate. Doxorubicin was co-loaded in the micelles through π-π stacking interaction with EGCG, which assured high stability of the formulation under physiological conditions and maximized the synergistic therapeutic effect between the chemotherapeutic drug and the bioflavonoid [11,39]. A similar synthesis procedure was adopted by Chung et al., who designed self-assembled micellar nanocomplexes through a two-step technology, comprising of core formation by complexation between oligomerised EGCG and the anticancer protein Herceptin, followed by subsequent shell synthesis through complexation between polyethylene glycol and epigallocatechin gallate [8].

**Figure 3:**
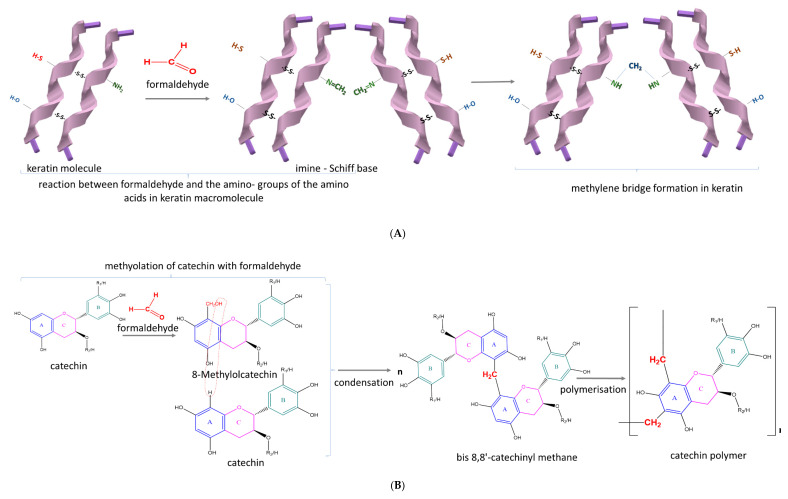

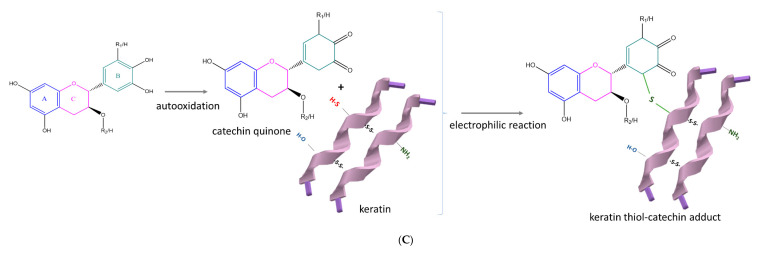
Synthesis mechanism of catechin-loaded keratin-based nanoparticles by: (**A**) methylene bridge formation in keratin, (**B**) catechin polymer synthesis by methyolation with formaldehyde, condensation and polymerization (**C**) electrophilic reaction of the thiol groups of keratin, with catechin quinone obtained by catechin autooxidation (Adapted from References [11,42,43,44]).

The one-pot synthesis strategy presented by Yi et al. [11] for the design of catechins-loaded keratin-based nanoparticles encompasses a multistage chemical process of methylolation, condensation and polymerisation. The aldol condensation reaction between catechins and keratins in the presence of formaldehyde resulted in the formation of a schiff-base, while the reaction between the imine and nucleophilic amino acid residues led to the formation of methylene-bridged oligomeric catechins (Figure 3) [11,42,43,44].

**Figure 4:**
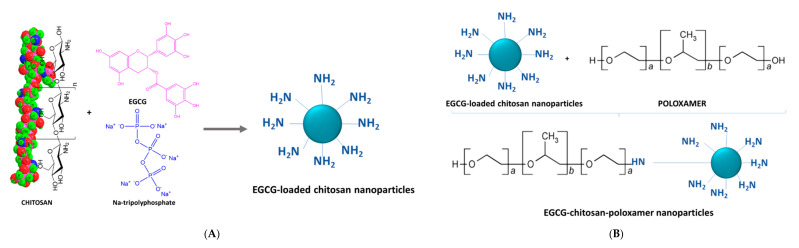

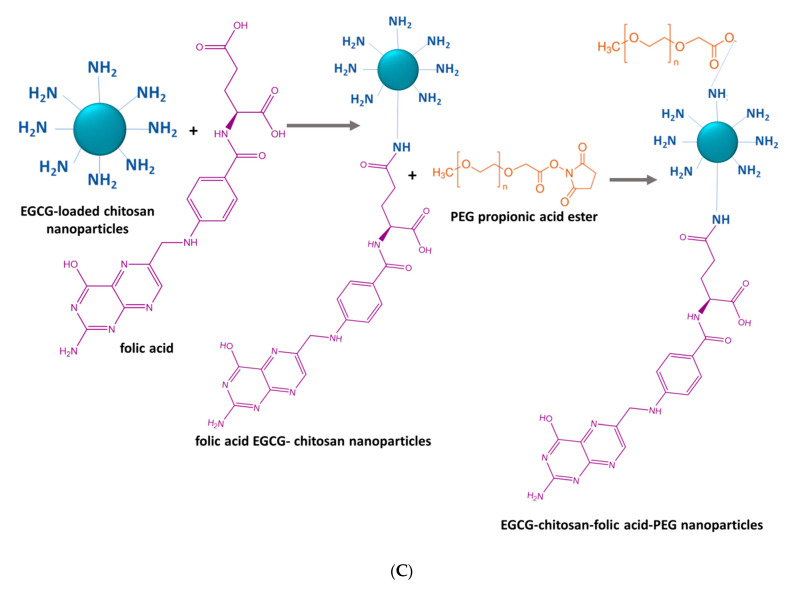
Synthesis mechanism of epigallocatechin gallate (EGCG)-loaded chitosan-folic acid-PEG and chitosan-poloxamer nanoparticles: (**A**) synthesis of EGCG-chitosan nanoparticles with Na-tripolyphosphate, (**B**) modification of EGCG-chitosan nanoparticles with poloxamer, (**C**) synthesis of EGCG-chitosan-folic acid-PEG nanoparticles via two consecutive reactions with folic acid and PEG propionic acid ester (Adapted from References [45,46,47]).

**Figure 5:**
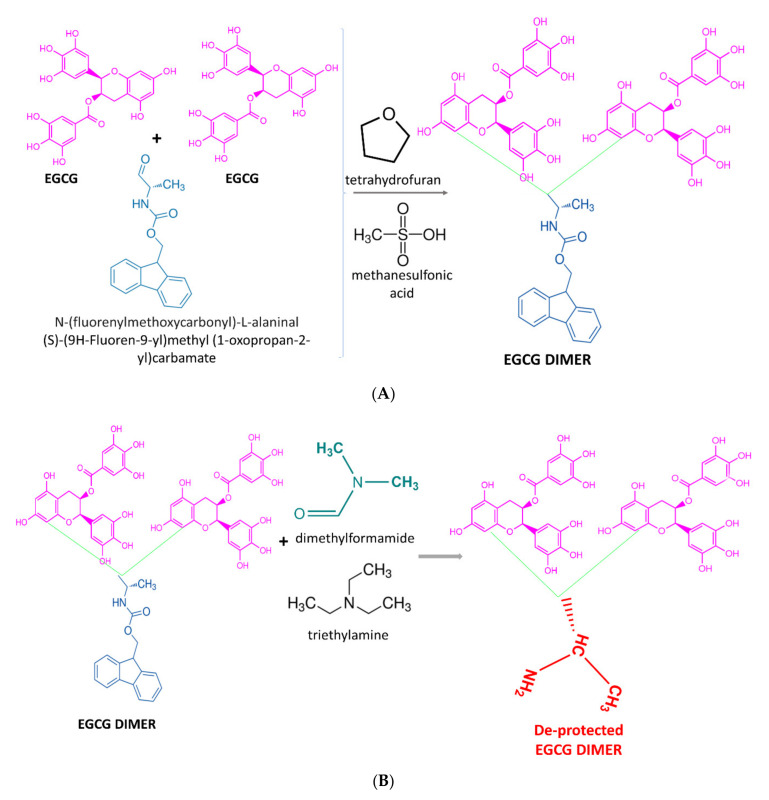

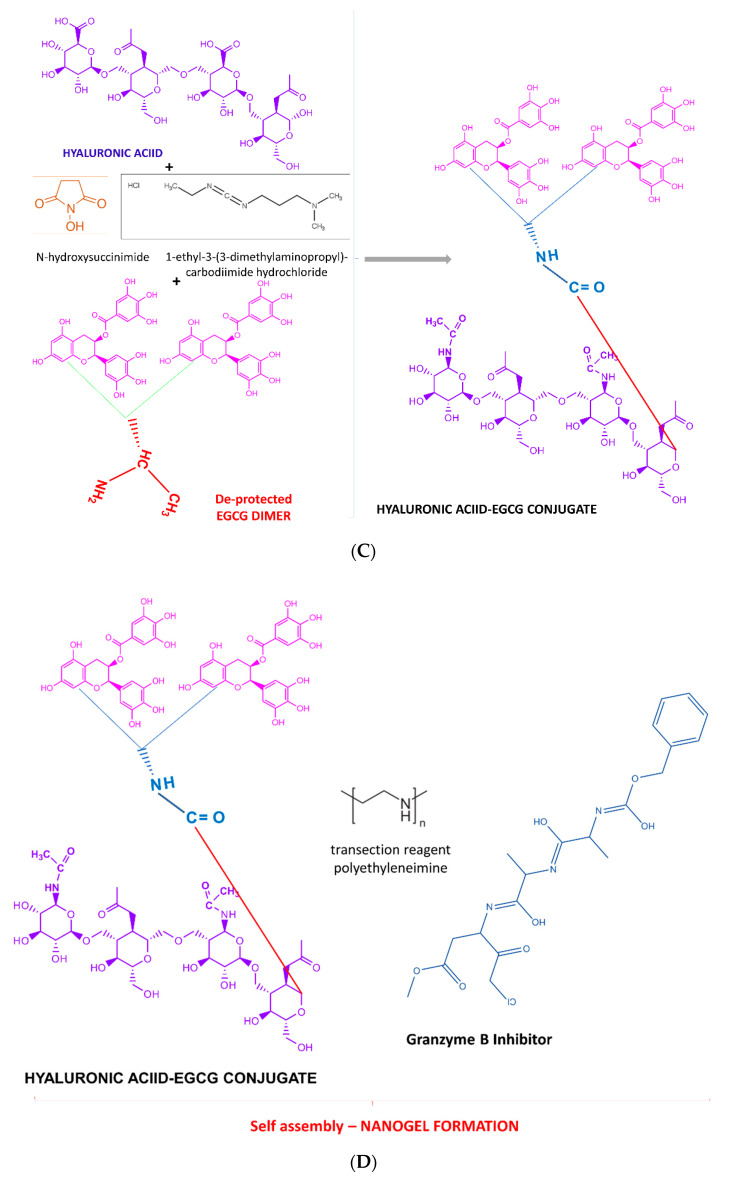
Synthesis mechanism of self-assembled hyaluronic acid-EGCG-polyethyleneimine-Granzyme B nanogel (Adapted from Reference [48]): (**A**) formation of EGCG dimer by an aldehyde-mediated reaction between the aldehyde N-(fluorenylmethoxycarbonyl)-L-alaninal and A-ring of EGCG molecules, (**B**) de-protection of EGCG dimer in the presence of dimethylformamide and trimethylamine, (**C**) conjugation of EGCG de-protected dimer to hyaluronic acid by a carbodiimide reaction, (**D**) nanogel formation by self-assembly between hyaluronic acid–EGCG conjugate, the transection reagent polyethyleneimine and Granzyme B Inhibitor.

EGCG-loaded chitosan-folic acid-polyethylene glycol (PEG) and chitosan-poloxamer nanoparticles were synthesized by a three-step procedure comprising of: (i) ionotropic gelation between chitosan amine groups and Na-tripolyphosphate groups, and EGCG attachment via covalent and H-bonding, (ii) amide formation reaction between folic acid-activated -COOH/poloxamer -OH groups and chitosan -NH_2_ groups, and (iii) conjugation of polyethylene glycol (PEG) to EGCG-chitosan-folic acid nanoparticles via reaction between ester group (-COOR) of succinimidyl ester of PEG propionic acid and chitosan -NH_2_ group (Figure 4) [45,46,47]. A similar general three-step synthesis concept was applied earlier by Liang et al. [48] for the production of hyaluronic acid–EGCG conjugates (Figure 5). The process stages included: (i) synthesis of EGCG dimer I via a reaction between EGCG, N-(9-fluorenylmethoxycarbonyl)-L-alanial (Fmoc-Ala) tetrahydrofuran and methylsulfonic acid, (ii) deprotection of dimer 1 in dimethylformamide and formation of EGCG dimer II and (iii) conjugation of dimer II onto hyaluronic acid by a carbodiimide reaction with 1-ethyl-3-(3-dimethylaminopropyl)-carbodiimide hydrochloride through activation of hyaluronic acid -COOH group with N-hydroxysuccinimide [48]. Finally, the novel ternary nanogel for targeted intracellular delivery of the serine preotease Granzyme B into cancer cells was formed by self-assembly between hyaluronic acid–EGCG conjugates, linear polyethylenimine and Granzyme B [48].

### 2.6. Direct Addition, Layer-by-Layer Assembly and Soaking Methods

The method of layer-by-layer (LbL) assembly is based on the construction of multilayers, constructed with nanometre precision and predetermined layer composition via sequentially saturating the adsorption of a layer of each component on a template surface. Among the most significant advantages of the layer-by-layer approach are multifunctionality and responsiveness to a multitude of stimuli [49]. Shutava et al. [50] applied this method for the preparation of EGCG gallate/gelatin layer-by-layer assembled films and microcapsules. It was established that since the EGCG content in the gelatin/polyphenol film material was high, encapsulation of EGCG in LbL assembled films and microcapsules can be a perspective way to obtain new formulations for drug delivery applications [50].

Although not so technologically complicated and specific from a biochemical engineering aspect, direct addition and soaking methods (swelling, absorption) have been widely applied and elaborated in the recent decade. They provided catechin-delivery systems with high entrapment capacity, prolonged and targeted in vitro release, significant bioactivity and even synergistic therapeutic activity (Table 1). Since the EGCG content in the gelatin/polyphenol film material was rather high (up to 30% *w*/*w*), encapsulation of EGCG in LbL assembled films and microcapsules could be accepted as a perspective way to obtain new formulation of this cancer chemo-preventive polyphenol for drug delivery applications.

## 3. Modes of In Vitro Catechins Release

Efficient delivery of nutraceuticals depends on the release mechanism and rate, as well as on the behaviour of the biocarrier and the encapsulated bioactive agent in physiological medium [64,65,66]. With respect to sustained release, the successful therapeutic treatment requires specific therapies [23,24,25,26,27] associated with the achievement of either temporal, or distribution-controlled release, which ensures delivery over an extended/specific time period into the systemic circulation system or targeted release to specific active sites, respectively [4,67]. This behaviour ensures the elimination of undesirable “burst effects” and provides prolonged and controlled release. Moreover, maintaining the therapeutic drug concentration for the desired period is even more significant from a clinical aspect and outlines a demanding area of drug delivery science [68].

The most widely applied analytical methods for quantification of catechins released from biopolymer carriers include: ultraviolet-visible (UV/Vis) spectrophotometry, high-performance liquid chromatography (HPLC) [14,16,60], colorimetric methods [22] and differential pulse voltammetry [32]. The concentrations of bioflavonoids solutions (in EtOH, double distilled water, simulated gastrointestinal medium) were determined in the UV spectral region at maximum wavelengths λ = 230, 273, 274 and 275 nm for EGCG [14,36,61], and 276 and 280 nm for catechin [27,63]. The concentrations of EGCG in biological samples was determined by liquid chromatography tandem mass spectrometry (LC-MS/MS) analysis [55].

**Figure 6:**
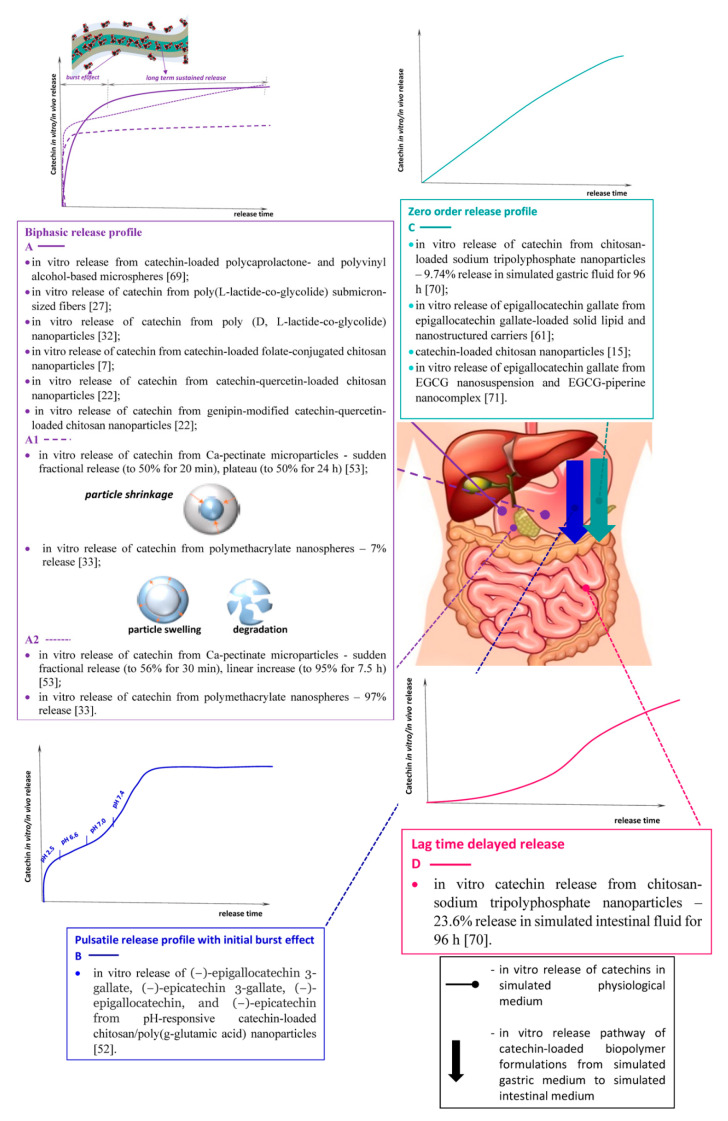
Common in vitro release profiles and release capacity of various catechins-loaded biopolymer matrices in simulated gastrointestinal medium [7,15,22,27,32,33,52,53,61,69,70,71].

Catechins in vitro release kinetics from nano-/micro-biopolymer formulations depend on a number of factors: encapsulation mechanism, pH of the medium, types of host–guest interactions between the bioflavonoid molecules and the carrier, presence of other bioactive agents, physicochemical, structural and morphological characteristics, biochemical properties and biological activity of the biomatrix, etc. (Table 1). The values of these parameters are responsible for the mode of the release profile of the bioactive molecules from the carrier matrix in different simulated physiological media (Figure 6).

An in vitro release study of catechin-loaded polycaprolactone- and polyvinyl alcohol-based microspheres in phosphate buffered saline (PBS) (pH 7.4) medium exhibited a biphasic sustained release. The release profile encompassed a small initial burst region, followed by a long-term slow-release stage, which was regardless of the flavonoid loading extent [69]. Similar release behaviour was observed for catechin-loaded poly(l-lactide-co-glycolide) submicron-sized fibres [27] and nanoparticles [32], in PBS at pH 7.4, and in Britton-Robinson buffer (pH = 7.4, 4.5, or 2) respectively, as a function of flavonoid concentration, time and pH. The in vitro release study of Ghitescu et al. [27], combined with polymer degradation investigations, established a predominant diffusion-controlled release mechanism, which depicted marginal degradation of the carrier during the time span of catechin delivery. The activity of the released catechin was assessed for its influence on multi-walled carbon nanotubes-induced formation of reactive oxygen species (ROS) in the human alveolar epithelial the cell line A549 [27]. Pool et al. [32,33] applied the advantageous differential pulse voltammetry technique. It allowed real-time monitoring of catechin release from polymeric nanoparticles and reduction of solvents amounts, thus lowering the experimental costs. The in vitro release profile (in Britton-Robinson buffer at pH = 7.4, 4.5, or 2) of catechin from PLGA particles was pH-dependant, which indicated faster release of the flavonoid in acidic medium [32]. Another simulated gastrointestinal study outlined that catechin was slowly released under gastric conditions (pH 2.5), but rapidly under small intestine conditions (pH 7.2) [33]. The in vitro release profiles of the bioflavanol from Ca-pectinate microparticles [53], folate-conjugated chitosan nanoparticles [7], polymethacrylate nanospheres [33], solid lipid and nanostructured lipid carriers [55] in simulated gastric and intestinal fluid also followed a bimodal kinetics curve. According to Lee et al. [53], in acidic medium, the release kinetics curve of Ca-pectinate microparticles is characterised with a steep initial increase, followed by a horizontal plateau. The observed approximately 50% release efficiency was attributed to shrinkage of Ca-pectinate gel structure, resulting in reduced bioflavonoid release. In alkaline intestinal fluid, the release profile comprised of a commensurable initial burst effect region and subsequent smooth linear section up to ~95% due to microparticles’ swelling and subsequent degradation [53]. The concept of Pool et al. [33] explained the analogical deviations of catechin release behaviour from polymethacrylate nanospheres in simulated gastric and intestinal fluids by alterations in the carboxylic groups’ ionic state in the polymer formulation. It suggested protonation of the carboxylic groups in acidic medium (−COOH_2_^+^), leading to reduced electrostatic repulsion forces between the polymer molecules, and de-protonation of the functional groups (−COO^−^) in alkaline medium. The latter induced strong electrostatic repulsion interactions, which in turn promoted particle swelling and dissociation [33]. With respect to the bimodal release curves, the initial burst effects were attributed to adsorbed residual catechin in the superficial zone or the shell surrounding the particle/fibre surface, while the prolonged zone was due to a release from a bioflavonoid-enriched rigid and/or hydrophobic core [27,53].

The study of Tang et al. established a modified pulsatile catechin release profile from self-assembled nanoparticles composed of chitosan and poly(g-glutamic acid) in simulated pH environments [52]. The kinetics release curve comprised of three major sections: (i) initial 20% burst release at pH 2.5 for 6 min, (ii) moderate (up to 40%) release at pH 6.6 (the duodenum) and (iii) complete release in jejunum, proximal ileum (pH 6.6–7.0) and distal ileum (pH 7.4). The proposed concept for the release mechanism in the last stage proposed nanoparticles’ infiltration into the mucus, resulting in gradual disintegration, facilitated catechin release and subsequent permeation through the paracellular pathway to the bloodstream [52]. Pulsatile drug delivery systems have some beneficial advantages such as drug delivery at the right time, at the right active site and in the right amount, which provides more benefits than conventional dosages and increased patient compliance. However, they suffer from the disadvantages of low drug loading capacity, incomplete drug release and multiple manufacturing steps. Thus, the investigations directed to the optimisation of the design and physiological behaviour of such drug delivery systems face challenges that have to be overcome [72,73,74].

Zero-order and delayed release are among the most prevalent conventional drug release mechanisms due to the specific advantages they offer. In general, hydrophilic matrices are capable of providing near zero-order release based on the molecular weight, formulation and geometry. The primary mechanism involves polymer swelling and subsequent formation of a gel layer, followed by drug release by dissolution, diffusion and/or erosion, hence facilitating zero-order release [75], whereas delayed drug release focuses on the protection of acid-sensitive drugs against gastric fluid and safeguarding gastric mucosa against aggressive actives [76]. EGCG in vitro release from solid-lipid and nanostructured carriers in simulated physiological (gastric and intestinal) media followed a double kinetics profile, which was characterised by: a stage of initial fast release, followed by a delayed release region. These stages were mathematically interpreted by the zero-order and Korsmeyer–Peppas models, respectively [61].

The limited number of studies subjected to the synthesis and analyses of efficient pulsatile, zero-order and delayed carrier systems, applicable for catechins delivery, outlines an important perspective area for future research.

## 4. Bioactivity Enhancements of Catechins-Loaded Biopolymer Formulations

### 4.1. Antioxidant Activity

**Figure 7:**
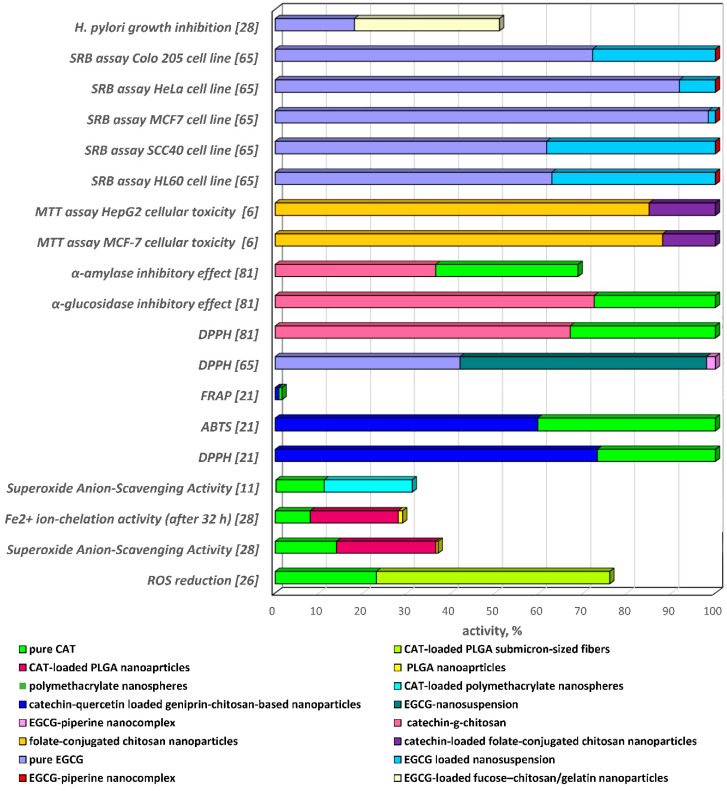
Biological activity of catechins-loaded bioformulations vs. pure bioflavonoids and biopolymers: comparison of the antioxidant, antiproliferative and antibacterial activity of pure catechin, pure epigallocatechin gallate, unloaded biopolymer formulations with the respective bioactivity of catechin- and epigallocatechin gallate-loaded biopolymer formulations (nanoparticles, nanofibers, nanosuspensions, nanocomplexes).

Reactive oxygen species (ROS) are common products of normal aerobic cellular metabolism, but increased ROS levels lead to oxidative stress and cellular damage. Therefore, effective antioxidant therapies are needed to prevent ROS overproduction [23,77,78]. Catechins are natural polyphenolic compounds that are widely utilised as nutraceuticals for improving antioxidant, anti-inflammatory, anti-tumour, neuroprotective, antibacterial activities, etc. The main obstacles for their efficient therapeutic applications, however, arise from restricted bioactive performance due to low oral bioavailability, low permeability, reduced solubility, insufficient gastric residence time and digestive instability. In the last few decades, innovative scientific studies have presented effective solutions for overcoming these limitations. These investigations are based on the development and integrative laboratory and clinical analyses of catechins-loaded biopolymer formulations and assessment of their bioactivities (Figure 7) [70,79,80].

The study of Ghitescu et al. established that catechin released from PLGA submicron-sized fibres in A549 cells reduced multi-walled carbon nanotube-induced ROS signal by ~50%, as compared to the 23% ROS reduction activity of pure catechin [23]. The ferric reducing ability of plasma (FRAP) values determined in vivo in a rat model after feeding the experimental animals with catechin-loaded Ca-pectinate microparticles were significantly higher than the corresponding values obtained from rats fed with free catechin. The maintenance of the bioflavonoid antioxidant activity was attributed not only to its sustained in vivo release into the plasma but also to retardation of catechin elimination [53]. Tang et al. and Chen et al. reported retention of the antioxidant activity and protection of tea catechins by chitosan-poly(g-glutamic acid) and gelatin nanoparticles by the obtained sustained free radical (DPPH^•^ and ABTS^•+^) scavenging assays [52,58]. The established significant increase in the scavenging rate of free radicals with pH increase was attributed to improvement of the electron-donating ability upon deprotonation of the catechin aromatic hydroxyl group [52]. Besides, catechin-loaded gelatin nanoparticles exhibited 28−41% enzymatic inhibition to trypsin against gelatin degradation [58].

According to Pool et al. and Dahiya et al., the inhibition of the action of free radicals and the chelating properties were enhanced when catechin [32] and EGCG [71] were encapsulated within PLGA nanoparticles. Catechin-loaded nanoparticles exhibited greater lipid oxidation inhibition, which could be attributed, on the one hand, to the protection of the nanoparticles during the incubation process and, on the other hand, to oxidation of free unprotected catechin by the molecular oxygen, formed by the stirring process, which provoked a decrease of its antiradical properties [32]. Similarly, EGCG nanosuspension and EGCG-piperine nanocomplex displayed significantly higher radical scavenging activity than pure EGCG [71]. The improvement in the superoxide anion scavenging activity (O_2_^•−^) of catechin, observed by Pool et al., encapsulated into polymethacrylate nanospheres (at concentration 35 μM), was attributed to the fact that it was in an amorphous state, which characterises with higher solubility and increased bioaccessibility, as compared to the free crystalline catechin [33]. Catechin-quercetin-loaded geniprin-modified chitosan-based nanoparticles manifested higher DPPH^•^ and ABTS^•+^ radicals scavenging activity as compared to the activity of the combined bioflavonoids. Besides, the DPPH^•^ and ABTS^•+^ scavenging activity of the composite formulation was comparable and FRAP values were lower than these for each of the polyphenols. The latter observations outlined the ability of the biopolymer nanoparticles to enhance and/or retain the antioxidant activity of catechin/quercetin combination [22]. Catechin-loaded Ca-pectinate beads demonstrated similar antioxidant behaviour—maintained FRAP scavenging activity in alkaline-simulated intestinal fluid and protection of catechin molecules from the alkaline environment [23]. Kaur et al. [15] explained the increased antioxidant capacity of chitosan nanoparticles loaded with hydrophilic drugs by the matrix structure of ionic polymers, which stabilises the bioactive compound. The higher and prolonged DPPH, NO and H_2_O_2_ scavenging activity of catechin-loaded nanoparticles in comparison with pure catechin was experimentally established [15]. OSA-starch, soybean lecithin and β-glucan-based solid micro-formulations loaded with EGCG also displayed improved antioxidant activity, which was determined by the oxygen radical absorbance capacity (ORAC) method. The experimental results proved 1.5-fold higher cellular antioxidant activity (Caco-2 cells) of EGCG-loaded lecithin and EGCG-loaded β-glucan microparticles than free EGCG at the same concentrations [56]. The transepithelial/transendothelial electrical resistance in vitro experiments of Tang et al. [52] established that chitosan poly(g-glutamicacid) nanoparticles had the potential to open the tight junctions between Caco-2 cells with a reversible effect on the tight junctions’ integrity. The latter donated to the increased radical scavenging activity of the nanoparticles loaded with catechins and for the intensified bioflavonoids transport between Caco-2 cell monolayers [52].

Samanta et al. [81] conducted in vivo experiments applying combined therapy with PLGA nanoparticles co-encapsulated with (+)-catechin hydrate and the chelator sodium meta borate to reduce the oxidative damage, provoked by fluoride-induced toxicity and mitochondrial impairment in experimental rats during chronic exposure to fluoride. The results displayed approximately two-fold reduction of fluoride levels in rat liver, brain and kidney [81].

**Figure 8:**
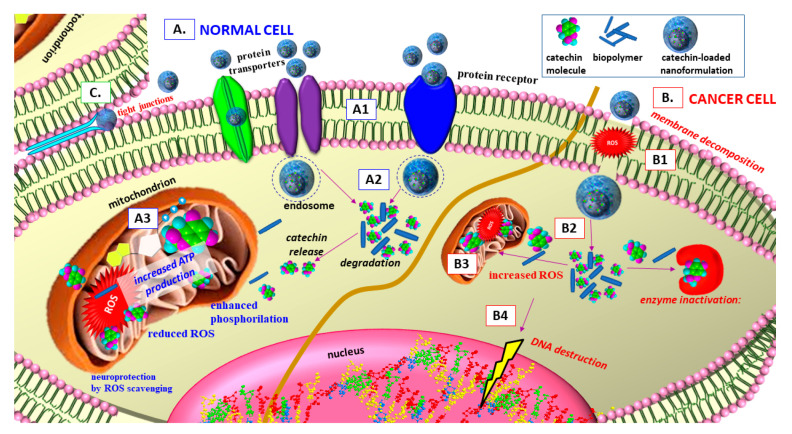
Catechins-loaded biopolymer nano formulations antioxidant/pro-oxidant/antiproliferative presumptive pathway and activity in intra- and inter-cellular environment of normal and cancer cells [7,11,22,52,58,71,94,95,101,107,108,109,110,111]: (**A**). In intracellular normal cell environment: (**A1**). Diffusive transport via protein transporters, (**A2**). Intracellular degradation of the bioflavonoid/biopolymer composite, subsequent catechins release, (**A3**). In mitochondrion environment. (**B**). In intracellular cancer cell environment: (**B1**). Entrance into the cancer cell, (**B2**). Intracellular degradation, subsequent catechins release, (**B3**,**B4**). Increased ROS production, (**B5**). Inactivation of enzymes. (**C**). In intercellular environment: opening of tight junctions between cells with a reversible effect on the tight junctions’ integrity.

In conclusion, a stable tendency of increased antioxidant activity of catechins-loaded nano- and micro-biopolymer formulations over pure bioflavonoids, attributed to elevated stability of the encapsulated polyphenols within the carriers, especially in gastric fluids, was established in the recently cited scientific studies (Figure 7 and Figure 8). These observations are outright prerequisites for even more intensive future research and for the development of innovative, highly efficient bioflavonoid-delivery systems with improved antioxidant capacity as potential therapeutic agent candidates. However, due to the limited number of in vivo studies, further in vivo investigations are warranted to explicate the therapeutic efficacy of encapsulated catechins.

### 4.2. Antidiabetic Activity

Certain studies reported the potential of catechins to reduce blood glucose, body weight and body mass index (BMI) in both elderly and obese subjects by stimulating thermogenesis [82,83]. Besides, attenuation of diabetic autonomic neuropathy in streptozotocin-induced diabetic rats was observed [84,85,86,87]. These results are indicative of the antidiabetic capacity of the bioflavonoids group, although according to Lankatillake et al. [88], the lack of standard protocols for exploring the antidiabetic properties of plant extracts has been outlined as an obstacle. Thus, the development of standardised testing methods for known therapeutic targets of diabetes are necessary and beneficial [88].

Nevertheless, few in vitro/in vivo studies have recently confirmed the defence that catechins encapsulated in biopolymer carriers can offer against diabetes (Figure 7). In this respect, in vivo investigations with rats treated with catechin-loaded Eudragit microparticles established a significant reduction of blood glucose, cholesterol, low-density lipoprotein (LDL), free fatty acid and triglyceride concentrations in comparison to rats treated with pure catechin. Absence of obesity after a 60-day treatment period was also determined [54]. The earlier study of Zhu and Zhang [89] reported potent α-glucosidase and moderate α-amylase inhibitory effects of catechin-grafted-chitosan as compared to pure catechin and chitosan. This effect was attributed to a probable synergism and indicated the potential of the bioflavonoid carrier system for type 2 diabetes treatment [89].

The limited knowledge on the antidiabetic activity of catechins-delivery systems combined with the recently achieved scientific expertise on catechins bioactivity outline this research aspect as a new challenge in front of the scientific society.

### 4.3. Antiproliferative Activity

According to published evidence, overproduction of ROS and the consecutive disruption of intracellular redox homeostasis are implicated in the processes associated with activation of signal pathways, leading to uncontrolled cell proliferation and carcinogenesis [90]. Due to their oxidative properties, ROS have caused damage of cellular macromolecules, including DNA damage, and initiated malignant conversion through mutation in proto-oncogenes, tumour suppressor genes and subsequent activation of signal transduction pathways, which are capable to maintain uncontrolled proliferation in damaged cells [91,92,93].

Experimental data have indicated that catechin encapsulated into different matrices could affect cellular proliferative activity specific to cancer cells and assist in the induction of cancer cell death. Moreover, released catechin could contribute to a decrease of the toxic side effects of conventional chemotherapeutic drugs, which have been applied in cancer therapy [94].

In this respect, Liu et al. [7] established that folate-conjugated chitosan nanoparticles exhibited little or no cytotoxicity even at low concentration toward MCF-7 and HepG2 cells, while the catechin-loaded nano-formulation displayed significant antiproliferative effects in a dose-dependent mode. The enhanced cytotoxic effect was attributed to the binding of the catechin-loaded nanoparticles with folate receptors on MCF-7 cells, and to subsequent improvement of the intracellular uptake through receptor-mediated endocytosis [7]. The study of Bae et al. [95] established that hyaluronic acid–cisplatin complexes exhibited high toxicity to cancer and normal cells, while hyaluronic acid–green tea catechin micellar nanocomplexes exerted differential cancer cell-killing effects by delivering their payload into CD44-overexpresssing cancer cells in a target-specific manner. From a pharmacokinetics aspect, the bioflavonoid-loaded nanocomplexes manifested prolonged circulation and enhanced tumour accumulation by intravenous administration [95]. Sana et al. reported, in their preclinical study, that functionalisation of PLGA nanocarriers with small ligand molecules, with high affinity toward prostate specific membrane antigen, significantly enhanced the anticancer potential of EGCG in vivo [83]. The screening of the anticancer effect against HL60, SCC40, MCF7, HeLa and Colo205 cell lines using the sulforhodamine B (SRB) assay of EGCG, EGCG nanosuspension and EGCG-piperine nanocomplex encapsulated in protein nanocarrier, outlined enhanced activity of the EGCG-loaded nano-formulations, as compared to the pure bioflavonoid in a dose-dependent manner. The increased biological activity of the nanocomplex was explained by the probable synergistic effect between EGCG and piperine [71]. According to the study of Haratifar et al. [9] the binding of EGCG to casein micelles did not affect the bioefficacy of EGCG and cell uptake at concentrations higher than 0.03 mg EGCG/mL skim milk. Cell proliferation data displayed that the EGCG–milk complexes were able to significantly decrease the proliferation of HT-29 cancer cells in a manner similar to that of free EGCG [9]. The in vitro and in vivo anticancer activity study of biocompatible, multifunctional and size-tunable nanoparticles based on naturally reproducible catechins, formaldehyde and keratin, confirmed that doxorubicin-loaded nanoparticles displayed enhanced effects in cancer treatments with much less systemic toxicity than free doxorubicin [11]. Other in vivo studies in xenograft mice established the effective stability and activity of EGCG nanoparticles against stomach, prostate and melanocyte carcinoma [96,97,98]. In addition, EGCG combined with cisplatin in a nanoparticulate formulation was developed as a new synergistic therapy for some invasive cancers [99,100].

The present review demonstrated evidence of the concept of the feasibility of catechins-loaded biopolymer formulations as effective therapeutic agents for cancer treatment (Figure 7 and Figure 8) and supported the hypothesis of their target-specific enhanced bioavailability and limited unwanted toxicity, outlining the potential of the systems for probable clinical outcomes [71,94].

### 4.4. Antibacterial Activity

Catechin/Zn complex-loaded *β*-chitosan nanoparticles displayed higher antibacterial activity against *E. coli* and *L. innocua*. The easier cells’ permeation of the smaller particles leads to sustained release of intracellular substances. As a result, due to the interrupted synthesis of cell membranes and intracellular proteins, and the subsequent death of bacterial strains, they exhibited higher antioxidant activity as compared to the larger nanoparticles. The minimum inhibitory concentration (MIC) and the minimum bactericidal concentration (MBC) of the smallest catechin/Zn-complex/chitosan nanoparticles against *L. innocua* and *E. coli* were 0.031 and 0.063 mg/mL, and 0.063 and 0.125 mg/mL, respectively [21]. Similar tendency of increased antibacterial activity of catechin/quercetin-loaded chitosan nanoparticles over the pure bioactive substances and unloaded nanoparticles against *Staphylococcus aureus*, *Bacillus subtilis* and *Escherichia coli* was established by Li et al. [7]. The positive surface charge of the bioflavonoids-loaded nanocarriers facilitated the interactive action of nanoparticles with the negatively charged cell membranes of most bacteria. This mechanism was outlined as a probable explanation of the observed bioactivity [22]. An in vivo study on *H. pylori* growth inhibition and AGS cell line viability established that EGCG-loaded fucose–chitosan/gelatin nanoparticles more significantly inhibited *H. pylori* growth and more effectively reduced *H. pylori*-associated gastric inflammation in a gastric-infected mouse model as compared to pure EGCG. The increased inhibitory effect of the bioflavonoid-carrier nano-formulation was attributed to the prolonged interaction of the hydrophilic negatively charged surface of the lipopolysaccharide outer membrane of *H. pylori* bacteria with the positively charged surface of EGCG-loaded nanoparticles [101]. Green tea catechins/ciprofloxacin-loaded nanoemulsion was tested for its antibacterial activity on resistant representative isolates of *E. coli*, *K. pneumonia*, *P. aeruginosa* and *P. mirabilis*. The results of the study confirmed the potential of the nano-formulations for application against extended spectrum β-lactamase- and metallo-β-lactamase-producing bacterial strains producing uropathogens. Pharmacokinetic parameters were investigated in the blood and target organs like kidney, urinary bladder and spleen in Sprague Dawley rats after oral and intravaginal administration of the nano-formulation. The results revealed that the radiolabelled nanoemulsion penetrated vaginal mucus rapidly and reached the target organs such as kidney and urinary bladder. The percent per gram of radiolabelled drug reaching the target organs was significantly higher via the intravaginal route [102].

Consequently, the major factors responsible for the improved antibacterial activity of catechins-loaded delivery systems, in comparison to the pure bioflavonoids, are the mucoadhesivity of the formulations, the extent of the electrostatic interactions between bacterial and nanoparticles’ surfaces, as well as the antibacterial capacity of the unloaded biopolymer nanocarriers (Figure 7).

### 4.5. Neuroprotective Activity

Added attention has recently been paid towards investigations on the neuroprotective activity of catechins [103,104], but there are few studies that encounter the neuroprotective effect of catechins-loaded biopolymer nano-formulations [105]. The findings of Halevas et al. [106] revealed the overall bioactivity of catechin-loaded poly(ethyleneglycol) (PEG)/cetyltrimethylammonium bromide (CTAB)-modified silica nanoparticles under conditions of Cu(II)-induced oxidative stress in neuronal and glial cultures. It was established that the hybrid nanoparticles enhanced the in vivo antioxidant capacity of catechin by affecting Cu(II) toxicity, cell loss and neuronal morphological lesions in primary rat hippocampal cultures [106].

Later on, Mandal et al. designed polylactide-based biodegradable nanoparticles terminated with trehalose molecule or dopamine and loaded with catechin. It was reported that the bioflavonoids nanoparticles enter neuronal cells, inhibit polyglutamine aggregation responsible for Huntington’s disease, decrease oxidative stress and enhance cell proliferation against polyglutamine aggregates [63].

Thus, the scientifically established bioactivity profile of such newly arisen catechin-loaded nanomaterials projects the development of potential neuroprotective nanotechnologies and their applicability as alternative therapies for neurodegenerative diseases.

In summary, Figure 8 represents the general behaviour of catechins-loaded bioformulations in intracellular and intercellular environment, as well as some specific mechanisms of the natural polyphenols and biopolymers physiological activity in normal and cancer cells. In intracellular normal cell environment, the bioflavonoid-loaded biopolymer composite enters the cell via diffusive transport via protein transporters/receptors through the cell membrane lipid bilayer by adsorption, penetration or interactions with transcriptional factors. The intracellular degradation of the bioflavonoid/biopolymer composite object leads to subsequent catechins release. In mitochondrion environment, the biomolecules cause probable increased oxidative phosphorylation and ATP production, reduced ROS production, uncoupled respiratory capacity of mitochondrial function and/or preservation of membrane integrity. The result of these bioactivities provokes increased antioxidant activity (Figure 8A). In intracellular cancer cell environment, the catechin/biopolymer system enters the cancer cell due to cancer cell membrane bilayer decomposition due to increased ROS production or inactivation of transcription factors. The subsequent stage of the composite intracellular degradation results in subsequent catechins release. The biomolecules probably increase ROS production due to altered mitochondrial activity, which would lead to subsequent nucleus DNA destruction. The inactivation of enzymes (DNA methyltransferases, histone deacetylases) inhibits DNA synthesis. The consequence of these effects is apoptosis and/or inhibition of cancer cell proliferation (Figure 8B). In intercellular environment, some catechin-loaded formulations possess the potential to open tight junctions between cells with a reversible effect on the tight junctions’ integrity, which provokes donation for increased radical scavenging activity and/or enhanced bioflavonoids transport between cell monolayers (Figure 8C).

## 5. Synergism, Mode of Delivery and Safety Aspects

In general, combination therapy is accepted as more effective over monotherapy due to possible enhancement of the bioactive potential, the achievement of synergistic effects and reduced toxicity [27,47,51]. However, if based on simply mixed administration of various drugs, it may provoke distinct pharmacokinetic profiles of the applied therapeutic agents and an inconsistent in vivo biodistribution, leading to reduced synergistic effects. In the last decade, a number of studies reported maximised synergistic bioactivity of multiple bioactive substances-loaded nanoparticles applied as combination therapy systems, which released and delivered the bioflavonoids at specific target sites [39]. The results of Cheng et al. suggested that EGCG-based polyion complex micelles could effectively overcome doxorubicin-induced cardiotoxicity and multidrug resistance [39]. The combined therapeutic effects of catechins-based carrier and the protein drug Herceptin exhibited an increased anticancer effect as compared to the free protein [8]. According to Dahiya et al., piperine potentiates the activity of EGCG, and both EGCG and piperine encapsulated into a zein nanocarrier displayed a synergistic antioxidant effect [71]. The significantly increased antioxidant activity of ternary lactoferrin/hyaluronic acid EGCG complexes at pH 5 was attributed to the ability of the hydrogels to restrict the access of the free radicals generated in the aqueous phase to the trapped EGCG [112].

Other significant aspects during the design, the investigations and the studies of the applicability potential of catechins-loaded micro-/nano-formulations that has to be encountered are: (i) the compliance of the route of administration and the delivery mechanism of the hybrid system with the specific bioactivity target, and (ii) encountering the accompanying advantages, disadvantages and potential unwanted side effects. In this respect, it has to be emphasised that in many cases, systematic delivery is associated with high toxicity in vital organs and increased drug plasma levels, while local delivery leads to lower drug clearance of the body and high drug interstitial penetration [113].

In conclusion, based on the scientific opinion on the safety of green tea catechins provided by the European Food Safety Authority (EFSA) Panel on Food Additives and Nutrient Sources added to Food, the in vivo studies, clinical investigations and therapeutic administration of green tea catechins from dietary sources including preparations, such as food supplements and infusions, has to be strictly controlled due to observed adverse hepatotoxicity effects, associated with the natural bioflavonoids’ consumption. Thus, the Panel recommends determination of catechins dose–response of hepatotoxicity, as well as examination of inter- and intra-species variability [8,114,115,116,117,118,119].

## 6. Conclusions and Future Perspectives

Recent scientific studies outline the beneficial role of catechins in the prevention and protection against diseases caused by oxidative stress. However, the precise design and synthesis/preparation of flavanols-loaded biopolymer micro-/nano-formulations are of paramount importance in view of their pivotal medical and pharmaceutical application as delivery systems. Further precise and thorough exploration of the exact bioactivities and physiological action mechanisms from the micro-molecular and cellular, to the macro-organism level, is necessary through meticulous laboratory and clinical trials. Besides, these innovative carrier systems have to handle the constantly increasing requirements of modern medicine associated with overcoming the inadequacies of chemotherapeutic drugs, ensuring favourable pharmacokinetics, avoiding irreversible toxic side effects, assuring stimuli-responsiveness in disassembling at target active sites, securing no leakage of non-specific agents and providing optimal therapeutic doses. Thus, successful scientific studies of the 21st century have to outstrip the forthcoming evolutionary biomedical challenges via integrative and multidisciplinary in silico, in vitro and in vivo research approaches to achieve proper conceptual design and target physiological activity and optimum therapeutic effect of efficient catechins-biopolymer delivery systems.

## Figures and Tables

**Table 1:** Effect of encapsulation methodology on the encapsulation efficiency, in vitro release and bioactivity of catechins-loaded biopolymer formulations.

Formulation Type	Preparation Method, Characteristics	EE, LE, EC *	In Vitro Release	Biological Activity	Reference
calcium-alginate microparticles	swelling absorption uniform morphological properties of particles	EE = 10.6–51.6%	sustained release in simulated gastric and intestinal fluids	-	[51]
chitosan-poly(γ-glutamic acid) (γ-PGA) self-assembled nanoparticles	polyelectrolyte self-assembly method particle size 134.3–147.8 nm	LE = 13.8–23.5%	40% released in simulated gastric fluid	pH-responsive Sustained free radical antioxidant activity	[52]
Ca-pectinate microparticles reinforced with liposome and hydroxypropylmethylcellulose (HPMC)	CaCl_2_ conc. 5.20%, HPMC conc. 0.08%, hardening time: 12.63 min, mean particle size: 88 µm	EE = 9.5–30.19%	in simulated gastric fluid: 50% constant release from 20 min to 24 h incubation period, in simulated intestinal fluid: 56% for the initial 30 min, up to about 95% for the next 7.5 h, complete release after 8 h incubation.	increased antioxidant activity in rat plasma	[53]
Eudragit RS 100 microparticles	-	EE = 92.3% Production yield = 63.46	-	in vivo antidiabetic efficacy	[54]
pH-sensitive polymeric nanoparticles	emulsion evaporation method, Poly(lactic-co-glycolic acid), lactic acid:glycolic acid, poly(vinyl alcohol, particle size = 91.3 ± 0.8 nm	EE = 80.8%	total released amount ~68% for 24 h	increased bioavailability of EGCG, lower proteinuria excretion of nephrotic syndrome in rats, lower kidney pathology scores	[55]
octenyl succinic anhydride (OSA)-starch, soybean lecithin and β-glucan solid microformulations	Gas-saturated solution drying	EE ≈ 80.5%		improved cellular antioxidant activity, improved storage stability, preservation of catechin bioactive properties	[56]
starch-based nanoparticles	ultrasonic homogenization, horse chestnut: particle size 322.7 nm water chestnut: particle size 559.2 nm lotus stem: particle size: 615.6 nm zeta potential: (−21.5)–(−18.05) mV	EE: 59.09% EE: 48.30% EE: 55.00% encapsulation offers thermal protection of catechin	release after 4 h: 4.8 mg/100 mg 3.39 mg/100 mg 5.35 mg/100 mg	retained bioactive properties during in vitro digestion	[57]
self-assembled tea catechin/gelatin nanoparticles	direct mixing, mean particle size 200 nm, zeta potential < 0			antioxidant activity, protection against enzymatic degradation	[58]
Ca-pectinate gel beads entrapping catechin-loaded liposomes	with/without hydroxypropylmethyl cellulose	EE decreased by 40–50% with 2–6%. CaCl_2_ concentration increase in gelling media		sustained oral delivery of catechins	[23]
(−)-epicatechin-loaded lecithin–chitosan–**D**-alpha-tocopheryl polyethylene glycol succinate (TPGS) nanoparticles	molecular self-assembly	LE 3.42 ± 0.85% EE 56.1 ± 3.9%		inhibitory effect on human breast cancer cell line	[59]
chitosan nanoparticles	mean particle size < 500 nm	EC = 4 µg/mg		increased bioavalability significantly enhanced in vitro intestinal absorption, stabilisation of catechins in the donor chamber	[60]
solid lipid nanocarrier (SLN) nanostructured lipid carrier (NLC)	high-shear homogenisation and ultrasonication technique, unloaded particles size: SLN—206 nm, NLC—216 nm epigallocatechin gallate loaded particles size: SLN 364 nm, NLC 300 nm	EE (SLN) = 83% EE (NLC) = 90%	in simulated gastric medium: burst release, at intestinal conditions: slower, sustained release	low toxicity, biocompatibility, potential for oral preventive and therapeutic supplementation	[61]
solid lipid nanoparticles	spherical nanoparticles hydrodynamic diameter: 300.2 nm	EE = 81%	slow, sustained release, Fickian diffusion	improved bioavailability, protection from degradation, no acute or sub-chronic toxicity, suitable for oral administration	[62]
polylactide-based biodegradable nanoparticle	Polylactide is terminated with anti-amyloidogenic trehalose molecule or neurotransmitter dopamine and resultant nanoparticle is loaded with antiamyloidogenic catechin molecule nanoparticles, homogeneous morphology, average size ~180–220 nm	LE = 50% LC = 6%	In vitro release 80% for 35 h	biocompatibility, biodegradability, enhanced cellular/extracellular availability multivalent interaction	[63]
catechin-quercetin-loaded chitosan nanoparticles genipin-modified catechin-quercetin-loaded chitosan nanoparticles	mean diameter: 180.4 ± 3.1 nm 190.7 ± 2.8 nm ellipsoidal shape decreased zeta potential no particle aggregation	EE = 76.35% ± 1.37%	Release 63% in PBS for 80 h Release 76% in PBS for 80 h	antioxidant and antibacterial activity	[22]
catechin-loaded chitosan nanoparticles	average particle size 68.76 nm resistant to aggregation	EE = 60.42%	release 82.56%	enhanced antioxidant activity, no significant toxic effect	[15]

* EE—encapsulation efficiency, LE—loading efficiency, EC—encapsulation capacity.

## References

[1] Sawicka B., Ziarati P., Krochmal-Marczak B., Skiba D. (2019). Nutraceuticals in food and pharmacy. Rev. Agron. Sci..

[2] Cai Z.Y., Li X.M., Liang J.P., Xiang L.P., Wang K.R., Shi Y.L., Yang R., Shi M., Ye J.H., Lu J.L. (2018). Bioavailability of tea catechins and its improvement. Molecules.

[3] Ye J.H., Augustin M.A. (2019). Nano- and micro-particles for delivery of catechins: Physical and biological performance. Crit. Rev. Food Sci. Nutr..

[4] Santos A.M.J.B., Tayo L.L. (2018). Synthesis of poly (lactic-co-glycolic acid) microspheres loaded with (−)-epigallocatechin-3-gallate-β-cyclodextrin inclusion complex using double solvent emulsification. AIP Conference Proceedings.

[5] Helal N.A., Eassa H.A., Amer A.M., Eltokhy M.A., Edafiogho I., Nounou M.I. (2019). Nutraceuticals’ Novel Formulations: The Good, the Bad, the Unknown and Patents Involved Involved. Recent Pat. Drug Deliv. Formul..

[6] Yaneva Z., Ivanova D., Nikolova N., Tzanova M. (2020). The 21st century revival of chitosan in service to bio-organic chemistry. Biotechnol. Biotechnol. Equip..

[7] Liu B., Wang Y., Yu Q., Li D., Li F. (2018). Synthesis, characterization of catechin-loaded folate-conjugated chitosan nanoparticles and their anti-proliferative effect. CyTA J. Food.

[8] Chung J.E., Tan S., Gao S.J., Yongvongsoontorn N., Kim S.H., Lee J.H., Choi S.H., Yano H., Zhuo L., Kurisawa M. (2014). Self-assembled micellar nanocomplexes comprising green tea catechin derivatives and protein drugs for cancer therapy. Nat. Nanotechnol..

[9] Haratifar S., Meckling K.A., Corredig M. (2014). Antiproliferative activity of tea catechins associated with casein micelles, using HT29 colon cancer cells. J. Dairy Sci..

[10] Scholl C., Lepper A., Lehr T., Hanke N., Schneider K.L., Brockmoeller J., Seufferlein T., Stinglet J.K. (2018). Population nutrikinetics of green tea extract. PLoS ONE.

[11] Yi Z., Sun Z., Chen G., Zhang H., Ma H., Su W., Cui X., Li X. (2018). Size-controlled, colloidally stable and functional nanoparticles based on the molecular assembly of green tea polyphenols and keratins for cancer therapy. J. Mater. Chem. B.

[12] Yaneva Z., Georgieva N., Staleva M. (2016). Development of d,l-α-tocopherol acetate/zeolite carrier system: Equilibrium study. Mon. Chem.-Chem. Mon..

[13] Lyubenova Yaneva Z. (2019). Nonsteroidal anti-inflammatory drug solid-state microencapsulation on green activated carbon—Mass transfer and host-guest interactions. Chem. Biochem. Eng. Q..

[14] Haratifar S., Meckling K.A., Corredig M. (2014). Bioefficacy of tea catechins encapsulated in casein micelles tested on a normal mouse cell line (4D/WT) and its cancerous counterpart (D/v-src) before and after in vitro digestion. Food Funct..

[15] Kaur R., Rajput R., Nag P., Kumar S., Rachana Singh M. (2017). Synthesis, characterization and evaluation of antioxidant properties of catechin hydrate nanoparticles. J. Drug Deliv. Sci. Technol..

[16] Szekalska M., Sosnowska K., Czajkowska-Ko’snik A., Winnicka K. (2018). Calcium chloride modified alginate microparticles formulated by the spray drying process: A strategy to prolong the release of freely soluble drugs. Materials.

[17] Sosnik A., Seremeta K.P. (2015). Advantages and challenges of the spray-drying technology for the production of pure drug particles and drug-loaded polymeric carriers. Adv. Colloid Interface Sci..

[18] Patel B.B., Patel J.K., Chakraborty S. (2014). Review of patents and application of spray drying in pharmaceutical, food and flavor industry. Recent Pat. Drug Deliv. Formul..

[19] Bagheri L., Madadlou A., Yarmand M., Mousavi M.E. (2014). Spray-dried alginate microparticles carrying caffeine-loaded and potentially bioactive nanoparticles. Food Res. Int..

[20] Santa-Maria M., Scher H., Jeoh T. (2012). Microencapsulation of bioactives in cross-linked alginate matrices by spray drying. J. Microencapsul..

[21] Zhang H., Jung J., Zhao Y. (2016). Preparation, characterization and evaluation of antibacterial activity of catechins and catechins–Zn complex loaded β-chitosan nanoparticles of different particle sizes. Carbohydr. Polym..

[22] Li F., Jina H., Xiao J., Yin X., Liu X., Lia D., Huang Q. (2018). The simultaneous loading of catechin and quercetin on chitosan-based nanoparticles as effective antioxidant and antibacterial agent. Food Res. Int..

[23] Lee J., Kim E., Chung D., Lee H.G. (2009). Characteristics and antioxidant activity of catechin-loaded calcium pectinate gel beads prepared by internal gelation. Colloids Surf. B Biointerfaces.

[24] Volf I., Ignat I., Neamtu M., Popa V.I. (2014). Thermal stability, antioxidant activity, and photo-oxidation of natural polyphenols. Chem. Pap..

[25] Pham Q.P., Sharma U., Mikos A.G. (2006). Electrospinning of polymeric nanofibers for tissue engineering applications: A review. Tissue Eng..

[26] Travis J.S., von Recum H.A. (2008). Electrospinning: Applications in drug delivery and tissue engineering. Biomaterials.

[27] Ghitescu P., Popa A., Schipanski A., Hirsch C., Yazgana G., Popa V.I., Rossi R.M., Maniura-Weber K. (2018). Catechin loaded PLGA submicron-sized fibers reduce levels of reactive oxygen species induced by MWCNT in vitro. Eur. J. Pharm. Biopharm..

[28] Hoseyni S.Z., Jafari S.M., Tabarestani H.S., Ghorbani M., Assadpour E., Sabaghi M. (2020). Production and characterization of catechin-loaded electrospun nanofibers from Azivash gum-polyvinyl alcohol. Carbohydr. Polym..

[29] Hoseyni S.Z., Jafari S.M., Tabarestani H.S., Ghorbani M., Assadpour E., Sabaghi M. (2021). Release of catechin from Azivash gum-polyvinyl alcohol electrospun nanofibers in simulated food and digestion media. Food Hydrocoll..

[30] Zemljič L.F., Maver U., Glaser T.K., Bren U., Hrnčič M.K., Petek G., Peršin Z. (2020). Electrospun Composite nanofibrous materials based on (poly)-phenol-polysaccharide formulations for potential wound treatment. Materials.

[31] Barreras-Urbina C.G., Ramírez-Wong B., López-Ahumada G.A., Burruel-Ibarra S.E., Martínez-Cruz O., Tapia-Hernández J.A., Félix F.R. (2016). Nano- and micro-particles by nanoprecipitation: Possible application in the food and agricultural industries. Int. J. Food Prop..

[32] Pool H., Quintanar D., Figueroa J.D., Marinho Mano C., Bechara J.E., Godínez L.A., Mendoza S. (2012). Antioxidant effects of quercetin and catechin encapsulated into PLGA nanoparticles. J. Nanomater..

[33] Pool H., Luna-Barcenas G., McClements D.J., Mendoza S. (2017). Development of polymethacrylate nanospheres as targeted delivery systems for catechin within the gastrointestinal tract. J. Nanopart. Res..

[34] Rodrigues S., Rosa da Costa A.M., Flórez-Fernández N., Torres M.D., Faleiro M.L., Buttini F., Grenha A. (2020). Inhalable spray-dried chondroitin sulphate microparticles: Effect of different solvents on particle properties and drug activity. Polymers.

[35] Peres I., Rocha S., Gomesa J., Morais S., Pereira M.C., Coelho M. (2011). Preservation of catechin antioxidant properties loaded in carbohydrate nanoparticles. Carbohydr. Polym..

[36] Paximada P., Echegoyen Y., Koutinas A.A., Mandal I.G., Lagaron J.M. (2017). Encapsulation of hydrophilic and lipophilized catechin into nanoparticles through emulsion electrospraying. Food Hydrocoll..

[37] Gadkari P.V., Balaraman M. (2015). Extraction of catechins from decaffeinated green tea for development of nanoemulsion using palm oil and sunflower oil-based lipid carrier systems. J. Food Eng..

[38] Huang Y.B., Tsai M.J., Wu P.C., Tsai Y.H., Wu Y.H., Fang J.Y. (2011). Elastic liposomes as carriers for oral delivery and the brain distribution of (+)-catechin. J. Drug Target..

[39] Cheng T., Liu J., Ren J., Huang F., Ou H., Ding Y., Zhang Y., Ma R., An Y., Liu J. (2016). Green tea catechin-based complex micelles combined with doxorubicin to overcome cardiotoxicity and multidrug resistance. Theranostics.

[40] Haraguchi I., Ooya T. Catechin-albumin conjugates: Potentials as an antioxidant-functionalized drug carrier. Proceedings of the 10th World Biomaterials Congress 2016.

[41] Sistanipour E., Meshkinia A., Oveisi H. (2018). Catechin-conjugated mesoporous hydroxyapatite nanoparticle: A novel nano-antioxidant with enhanced osteogenic property. Colloids Surf. B Biointerfaces.

[42] Takano T., Murakami T., Kamitakahara H., Nakatsubo F. (2008). Mechanism of formaldehyde adsorption of (+)-catechin. J. Wood Sci..

[43] Mori T., Ishii T., Akagawa M., Nakamura Y., Nakayama T. (2010). Covalent binding of tea catechins to protein thiols: The relationship between stability and electrophilic reactivity. Biosci. Biotechnol. Biochem..

[44] Liu J., Yong H., Yao X., Hu H., Yun D., Xiao L. (2019). Recent advances in phenolic–protein conjugates: Synthesis, characterization, biological activities and potential applications. RSC Adv..

[45] Safer A.M., Leporatti S., Jose J., Soliman M.S. (2019). Conjugation of EGCG and Chitosan NPs as a novel nano-drug delivery system. Int. J. Nanomed..

[46] Zeng L., Yan J., Luo L., Ma M., Zhu H. (2017). Preparation and characterization of (−)-Epigallocatechin-3-gallate (EGCG)-loaded nanoparticles and their inhibitory effects on human breast cancer MCF-7 cells. Sci. Rep..

[47] Zhou Y., Chen J.H., Wang H., Wang C.X., Zhang J.Y., Tao Y.W., Zheng G.D., Xie H.Y. (2011). Synthesis and characterization of folate-poly (ethylene glycol) chitosan graft-polyethylenimine as a non-viral carrier for tumor-targeted gene delivery. Afr. J. Biotechnol..

[48] Liang K., Ng S., Lee F., Lim J., Chung J.E., Lee S.S., Kurisawa M. (2016). Targeted intracellular protein delivery based on hyaluronic acid–green tea catechin nanogels. Acta Biomater..

[49] Delcea M., Möhwald H., Skirtach A.G. (2011). Stimuli-responsive LbL capsules and nanoshells for drug delivery. Adv. Drug Deliv. Rev..

[50] Shutava T.G., Balkundia S.S., Lvov Y.M. (2009). (−)-Epigallocatechin gallate/gelatin layer-by-layer assembled films and microcapsules. J. Colloid Interface Sci..

[51] Mandal R.S.K., Bhatt S., Jithin M.V., Lekshman A., Raguvaran R., Mondal D.B. (2019). Evaluation of encapsulated catechin in chitosan-sodium tripolyphosphate nanoparticle. J. Pharmacogn. Phytochem..

[52] Tang D.W., Yu S.H., Ho Y.C., Huang B.Q., Tsai G.J., Hsieh H.Y., Sung H.W., Mi F.L. (2013). Characterization of tea catechins-loaded nanoparticles prepared from chitosan and an edible polypeptide. Food Hydrocoll..

[53] Lee J.S., Kim H.W., Chung D., Lee H.G. (2009). Catechin-loaded calcium pectinate microparticles reinforced with liposome and hydroxypropylmethylcellulose: Optimization and in vivo antioxidant activity. Food Hydrocoll..

[54] Meena K.P., Vijayakumar M.R., Dwibedy P.S. (2017). Catechin-loaded Eudragit microparticles for the management of diabetes: Formulation, characterization and in vivo evaluation of antidiabetic efficacy. J. Microencapsul..

[55] Zhang G., Zhang J. (2018). Enhanced oral bioavailability of EGCG using pH-sensitive polymeric nanoparticles: Characterization and in vivo investigation on nephrotic syndrome rats. Drug Des. Dev. Ther..

[56] Gonçalves V.S.S., Poejo J., Matias A.A., Rodríguez-Rojo S., Coceroc M.J., Duarte C.M.M. (2016). Using different natural origin carriers for development of epigallocatechin gallate (EGCG) solid formulations with improved antioxidant activity by PGSS-drying. RSC Adv..

[57] Ahmad M., Mudgila P., Ganib A., Hamed F., Masoodi F.A., Maqsood S. (2019). Nano-encapsulation of catechin in starch nanoparticles: Characterization, release behavior and bioactivity retention during simulated in-vitro digestion. Food Chem..

[58] Chen Y.C., Yu S.H., Tsai G.J., Tang D.W., Mi F.L., Peng Y.P. (2010). Novel technology for the preparation of self-assembled catechin/gelatin nanoparticles and their characterization. J. Agric. Food Chem..

[59] Perez-Ruiz A.G., Ganem A., Olivares-Corichi I.M., García-Sánchez J.R. (2018). Lecithin–chitosan–TPGS nanoparticles as nanocarriers of (−)-epicatechin enhanced its anticancer activity in breast cancer cells. RSC Adv..

[60] Dube A., Nicolazzo J.A., Larson I. (2010). Chitosan nanoparticles enhance the intestinal absorption of the green tea catechins (+)-catechin and (−)-epigallocatechin gallate. Eur. J. Pharm. Sci..

[61] Frias I., Neves A.R., Pinheiro M., Reis S. (2016). Design, development, and characterization of lipid nanocarriers-based epigallocatechin gallate delivery system for preventive and therapeutic supplementation. Drug Des. Dev. Ther..

[62] Ramesh N., Mandal A.K.A. (2019). Pharmacokinetic, toxicokinetic, and bioavailability studies of epigallocatechin-3-gallate loaded solid lipid nanoparticle in rat model. Drug Dev. Ind. Pharm..

[63] Mandal S., Debnath K., Jana N.R., Jana N.R.R. (2020). Trehalose conjugated, catechin loaded polylactide nanoparticle for improved neuroprotection against intracellular polyglutamine aggregate. Biomacromolecules.

[64] Yaneva Z.L. (2018). Drug mass transfer mechanism, thermodynamics, and in vitro release kinetics of antioxidant-encapsulated zeolite microparticles as a drug carrier system. Chem. Biochem. Eng. Q..

[65] Yaneva Z., Georgieva N., Grumezescu A. (2018). Physicochemical and morphological characterization of pharmaceutical nanocarriers and mathematical modeling of drug encapsulation/release mass transfer processes. Nanoscale Fabrication, Optimization, Scale-up and Biological Aspects of Pharmaceutical Nanotechnology.

[66] Yaneva Z., Staleva M., Georgieva N. (2015). Study on the host-guest interactions during caffeine encapsulation into zeolite. Eur. J. Chem..

[67] Ruiz-Caro R., Veiga-Ochoa M.D. (2009). Characterization and dissolution study of chitosan freeze-dried systems for drug-controlled release. Molecules.

[68] Indurkhya A., Patel M., Sharma P., Abed S.N., Shnoudeh A., Maheshwari R., Deb P.K., Tekade R.K., Rakesh K.T. (2018). Influence of Drug Properties and Routes of Drug Administration on the Design of Controlled Release System. Dosage Form Design Considerations, Advances in Pharmaceutical Product Development and Research.

[69] Sivabalan M., Gayathri V., Kiruthika C., Madhan B. (2012). Formulation and evaluation of biodegradable polyphenolic microspheres for cancer. Int. J. Pharm. Technol..

[70] Samanta A., Bandyopadhyay B., Das N. (2016). Formulation of catechin hydrate nanocapsule and study of its bioavailability. Med. Chem..

[71] Dahiya S., Rani R., Dhingra D., Kumar S., Dilbaghi N. (2018). Conjugation of epigallocatechin gallate and piperine into a zein nanocarrier: Implication on antioxidant and anticancer potential. Adv. Nat. Sci. Nanosci. Nanotechnol..

[72] Jain D., Raturi R., Jain V., Bansal P., Singh R. (2011). Recent technologies in pulsatile drug delivery systems. Biomatter.

[73] Beugeling M., Grasmeijer N., Born P.A., van der Meulen M., van der Kooij R.S., Schwengle K., Baert L., Amssoms K., Frijlink H.W., Hinrichs W.L.J. (2018). The mechanism behind the biphasic pulsatile drug release from physically mixed poly(dl-lactic(-co-glycolic) acid)-based compacts. Int. J. Pharm..

[74] Gandhi S., Nuxoll E. (2016). Non-delaminating pulsatile release composites. Chem. Eng. Sci..

[75] Zhang L., Alfano J., Race D., Davé R.N. (2018). Zero-order release of poorly water-soluble drug from polymeric films made via aqueous slurry casting. Eur. J. Pharm. Sci..

[76] Montero Mirabet M., Skalsky B. (2017). Advanced Approaches for Delayed-Release Formulations. ONdrugDelivery Mag..

[77] Yaneva Z.L., Simeonov E.B., Georgieva D.I. (2020). In vitro Ultraviolet-B radiation mediated antioxidant response of Bulgarian Goldenrod (*Solidago virgaurea* L.) extract. Bulg. Chem. Commun..

[78] Rani V., Gupta K., Rani V., Yadav U. (2015). ROS in carcinogenesis and anticancerous drug-induced toxicity. Free Radicals in Human Health and Disease.

[79] Kailakua S.I., Mulyawantia I., Alamsyah A.N. (2014). Formulation of nanoencapsulated catechin with chitosan as encapsulation material. Procedia Chem..

[80] Dang S., Gupta S., Bansal R., Ali J., Gabrani R., Rani V., Yadav U. (2015). Nano-encapsulation of a natural polyphenol, green tea catechins: Way to preserve its antioxidative potential. Free Radicals in Human Health and Disease.

[81] Samanta A., Chanda S., Bandyopadhyay B., Das N. (2016). Establishment of drug delivery system nanocapsulated with an antioxidant (+)-catechin hydrate and sodium meta borate chelator against sodium fluoride induced oxidative stress in rats. J. Trace Elem. Med. Biol..

[82] Hashemipour M.A., Lotfi S., Torabi M., Sharifi F., Ansari M., Ghassemi A., Sheikhshoaie S. (2017). Evaluation of the effects of three plant species (*Myrtus communis* L., *Camellia sinensis* L., *Zataria multiflora* Boiss.) on the healing process of intraoral ulcers in rats. J. Dent..

[83] Pastoriza S., Mesías M., Cabrera C., Rufián-Henares J.A. (2017). Healthy properties of green and white teas: An update. Food Funct..

[84] Addepalli V., Suryavanshi S.V. (2018). Catechin attenuates diabetic autonomic neuropathy in streptozotocin induced diabetic rats. Biomed. Pharmacother..

[85] Grzesik M., Naparlo K., Bartosz G., Sadowska-Bartosz I. (2018). Antioxidant properties of catechins: Comparison with other antioxidants. Food Chem..

[86] Kempegowda P.K., Zameer F., Murar S.K. (2018). Delineating antidiabetic proficiency of catechin from Withania somnifera and its Inhibitory action on dipeptidyl peptidase-4 (DPP-4). Biomed. Res..

[87] Cosarca S., Tanase C., Muntean D.L. (2019). Therapeutic aspects of catechin and its derivatives—An update. Acta Biol. Marisiensis.

[88] Lankatillake C., Huynh T., Dias D.A. (2019). Understanding glycaemic control and current approaches for screening antidiabetic natural products from evidence-based medicinal plants. Plant Methods.

[89] Zhu W., Zhangba Z. (2014). Preparation and characterization of catechin-grafted chitosan with antioxidant and antidiabetic potential. Int. J. Biol. Macromol..

[90] Ivanova D., Zhelev Zh Aoki I., Bakalova R., Higashi T. (2016). Overproduction of reactive oxygen species—Obligatory or not for induction of apoptosis by anticancer drugs. Chin. J. Cancer Res..

[91] Collin F. (2019). Chemical basis of reactive oxygen species reactivity and involvement in neurodegenerative diseases. Int. J. Mol. Sci..

[92] Thrachootam D., Lu W., Ogasawara M.A., Nilsa R.D.V., Huang P. (2008). Redox regulation of cell survival. Antioxid. Redox Signal..

[93] Coleman W.B., Tsongalis G.T. (2006). Molecular mechanisms of human carcinogenesis. EXS.

[94] Sanna V., Singh C.K., Jashari R., Adhami V.M., Chamcheu J.C., Rady I., Sechi M., Mukhtar H., Siddiqui I.A. (2017). Targeted nanoparticles encapsulating (−)-epigallocatechin-3-gallate for prostate cancer prevention and therapy. Sci. Rep..

[95] Bae K.H., Tan S., Yamashita A., Ang W.X., Gao S.J., Wang S., Chung J.E., Kurisawa M. (2017). Hyaluronic acid-green tea catechin micellar nanocomplexes: Fail-safe cisplatin nanomedicine for the treatment of ovarian cancer without off-target toxicity. Biomaterials.

[96] Lin Y.H., Chen Z.R., Lai C.H., Hsieh C.H., Feng C.L. (2015). Active targeted nanoparticles for oral administration of gastric cancer therapy. Biomacromolecules.

[97] Khan N., Bharali D.J., Adhami V.M., Siddiqui I.A., Cui H., Shabana S.M., Mousa S.A., Mukhtar H. (2014). Oral administration of naturally occurring chitosan-based nanoformulated green tea polyphenol EGCG effectively inhibits prostate cancer cell growth in a xenograft model. Carcinogenesis.

[98] Siddiqui I.A., Bharali D.J., Nihal M., Adhami V.M., Khan N., Chamcheu J.C., Khan M.I., Shabana S., Mousa S.A., Mukhtar H. (2014). Excellent antiproliferative and pro-apoptotic effects of -epigallocatechin-3gallate encapsulated in chitosan nanoparticles on human melanoma cell growth both in vitro and in vivo. Nanomed. Nanotechnol. Biol. Med..

[99] Singh M., Bhatnagar P., Mishra S., Kumar P., Shukla Y., Gupta K.C. (2015). PLGA-encapsulated tea polyphenols enhance the chemotherapeutic efficacy of cisplatin against human cancer cells and mice bearing Ehrlich ascites carcinoma. Int. J. Nanomed..

[100] Ernest U., Chen H.Y., Xu M.J., Taghipour Y.D., Asad M.H.H.B., Rahimi R., Murtaza G. (2018). Anti-cancerous potential of polyphenol-loaded polymeric nanotherapeutics. Molecules.

[101] Lin Y.H., Feng C.L., Lai C.H., Lin J.H., Chen H.Y. (2014). Preparation of epigallocatechin gallate loaded nanoparticles and characterization of their inhibitory effects on Helicobacter pylori growth in vitro and in vivo. Sci. Technol. Adv. Mater..

[102] Atinderpal K., Kapoor N., Gupta S., Tyag A., Sharma R.K., Ali J., Gabrani R., Dang S. (2018). Development and characterization of green tea catechins and ciprofloxacin-loaded nanoemulsion for intravaginal delivery to treat urinary tract infection. Indian J. Pharm. Sci..

[103] Singh N.A., Mandal A.K.A., Khan Z.A. (2016). Potential neuroprotective properties of epigallocatechin-3-gallate (EGCG). Nutr. J..

[104] Singh D., Hembrom S. (2019). Neuroprotective Effect of Flavonoids: A Systematic Review. Int. J. Aging Res..

[105] Ovais M., Zia N., Ahmad I., Khalil A.T., Raza A., Ayaz M., Sadiq A., Ullah F., Shinwari Z.K. (2018). Phyto-therapeutic and nanomedicinal approaches to cure Alzheimer’s Disease: Present status and future opportunities. Front. Aging Neurosci..

[106] Halevas E., Nday C.M., Salifoglou A. (2016). Hybrid catechin silica nanoparticle influence on Cu(II) toxicity and morphological lesions in primary neuronal cells. J. Inorg. Biochem..

[107] Mukhopadhyay P., Prajapati A.K. (2015). Quercetin in anti-diabetic research and strategies for improved quercetin bioavailability using polymer-based carriers—A review. RSC Adv..

[108] Vaiserman A., Koliada A., Zayachkivska A., Lushchak O. (2020). Nanodelivery of natural antioxidants: An anti-aging perspective. Front. Bioeng. Biotechnol..

[109] Bernatoniene J., Kopustinskiene D.M. (2018). The role of catechins in cellular responses to oxidative stress. Molecules.

[110] La X., Zhang L., Li Z., Li H., Yang Y. (2019). (−)-Epigallocatechin gallate (EGCG) enhances the sensitivity of colorectal cancer cells to 5-FU by inhibiting GRP78/NF-κB/miR-155-5p/MDR1 pathway. J. Agric. Food Chem..

[111] Shen W., Wang Q., Shen Y., Gao X., Li L., Yan Y., Wang H., Cheng Y. (2018). Green tea catechin dramatically promotes RNAi mediated by low-molecular-weight polymers. ACS Cent. Sci..

[112] Liu R., Yan X., Liu Z., McClements D.J., Liu F., Liu X. (2019). Fabrication and characterization of functional protein–polysaccharide–polyphenol complexes assembled from lactoferrin, hyaluronic acid and (−)-epigallocatechin gallate. Food Funct..

[113] Apon A., Kamble P. (2018). Systematic vs local drug delivery systems in the treatment of periodontal diseases—A review. World J. Adv. Sci. Res..

[114] Younes M., Aggett P., Aguilar F., Crebelli R., Dusemund B., Filipic M., Frutos M.J., Galtier P., Gott D., EFSA ANS Panel (EFSA Panel on Food Additives and Nutrient Sources added to Food) (2018). Scientific Opinion on the safety of green tea catechins. EFSA J..

[115] Sergi C.M. (2020). Epigallocatechin-3-Gallate toxicity in children: A potential and current toxicological event in the differential diagnosis with virus-triggered fulminant hepatic failure. Front. Pharmacol..

[116] Galati G., Lin A., Sultan A.M., O’Brien P.J. (2006). Cellular and in vivo hepatotoxicity caused by green tea phenolic acids and catechins. Free Radic. Biol. Med..

[117] Jimenez-Saenz M., Martinez-Sanchez Mdel C. (2006). Acute hepatitis associated with the use of green tea infusions. J. Hepatol..

[118] Mazzanti G., Di Sotto A., Vitalone A. (2015). Hepatotoxicity of green tea: An update. Arch. Toxicol..

[119] Wang D., Wang Y., Wan X., Yang C.S., Zhang J. (2015). Green tea polyphenol (−)-epigallocatechin-3-gallate triggered hepatotoxicity in mice: Responses of major antioxidant enzymes and the Nrf2 rescue pathway. Toxicol. Appl. Pharmacol..

